# Design of Nanostraws in Amine-Functionalized MCM-41 for Improved Adsorption Capacity in Carbon Capture

**DOI:** 10.1021/acs.energyfuels.3c01318

**Published:** 2023-07-26

**Authors:** Oluwole Ajumobi, Borui Wang, Azeem Farinmade, Jibao He, Julia A. Valla, Vijay T. John

**Affiliations:** † Department of Chemical & Biomolecular Engineering, Tulane University, 6823 St. Charles Avenue, New Orleans, Louisiana 70118, United States; ‡ Coordinated Instrumentation Facility, Tulane University, 6823 St. Charles Avenue, New Orleans, Louisiana 70118, United States; § Department of Chemical & Biomolecular Engineering, University of Connecticut, Storrs, Connecticut 06269, United States

## Abstract

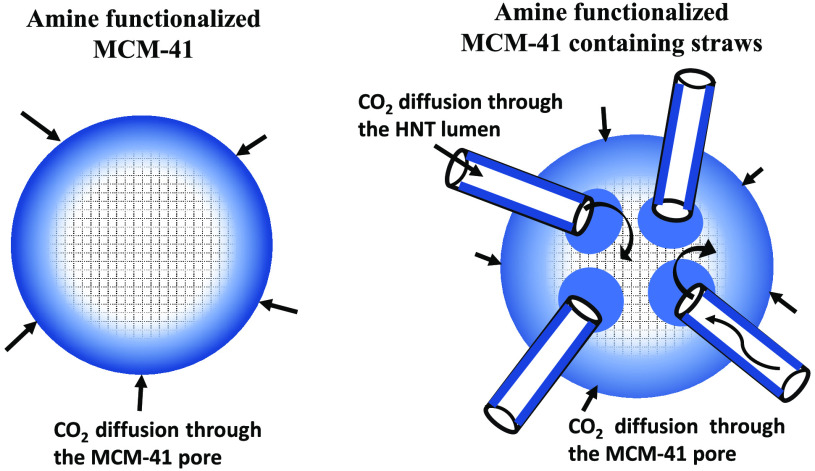

Polymeric amine encapsulation in high surface area MCM-41 particles for CO_2_ capture is well established but has the drawback of leaching out the water-soluble polymer upon exposure to aqueous environments. Alternatively, chemical (covalent) grafting amine functional groups from an alkoxysilane such as 3-aminopropyltriethoxysilane (APTES) on MCM-41 offer better stability against this drawback. However, the diffusional restriction exhibited by the narrow uniform MCM-41 pores (2–4 nm) may impede amine functionalization of the available silanol groups within the inner mesoporous core. This leads to incomplete amine functionalization and could reduce the CO_2_ adsorption capacity in such materials. Our concept to improve access to the MCM-41 interior is based on the incorporation of nanostraws with larger inner diameter (15–30 nm) to create a hierarchical porosity and enhance the molecular transport of APTES. Halloysite nanotubes (HNT) are used as tubular straws that are integrated into the MCM-41 matrix using an aerosol-assisted synthesis method. Characterization results show that the intrinsic structure of MCM-41 remains unaltered after the incorporation of the nanostraws and amine functionalization. At an optimal APTES loading of 0.5 g (*X* = 2.0), the amine-functionalized composite of MCM-41 with straws (APTES/M40H) has a 20% higher adsorption capacity than the amine-modified MCM-41 (APTES/MCM-41) adsorbent. Furthermore, the CO_2_ adsorption capacity APTES/M40H doubles that of APTES/MCM-41 when normalized based on the composition of MCM-41 in the composite particle with straws. The facile integration of nanostraws in MCM-41 leading to hierarchical porosities could be effective toward the mitigation of diffusional restriction in porous materials with potential for other catalytic and adsorption technologies.

## 1 Introduction

One of the major gases responsible for climate change is CO_2_^1^ generated in large quantities from fossil fuels and industrial activities.^2,3^ The industrial approach to remove CO_2_ involves the use of aqueous solvent-based amines, but the amine regeneration step has a high energy requirement due to energy wasted in solvent heating.^4^ This has led to the development of solid amine-based adsorbents where most of the energy costs are associated with regeneration of the amines.^5^ Common solid amine adsorbents are prepared by wet impregnation of polymeric amines within the pores of a porous support (class I adsorbents)^6^ or covalent grafting of amine functional groups onto the surfaces of a porous support (class II adsorbents).^7^ Class I adsorbents based on impregnation of the polymeric amines have the disadvantage of amine leaching under humid conditions, while class II adsorbents with grafted amines are stable under humid conditions.^8^

**Figure 1:**
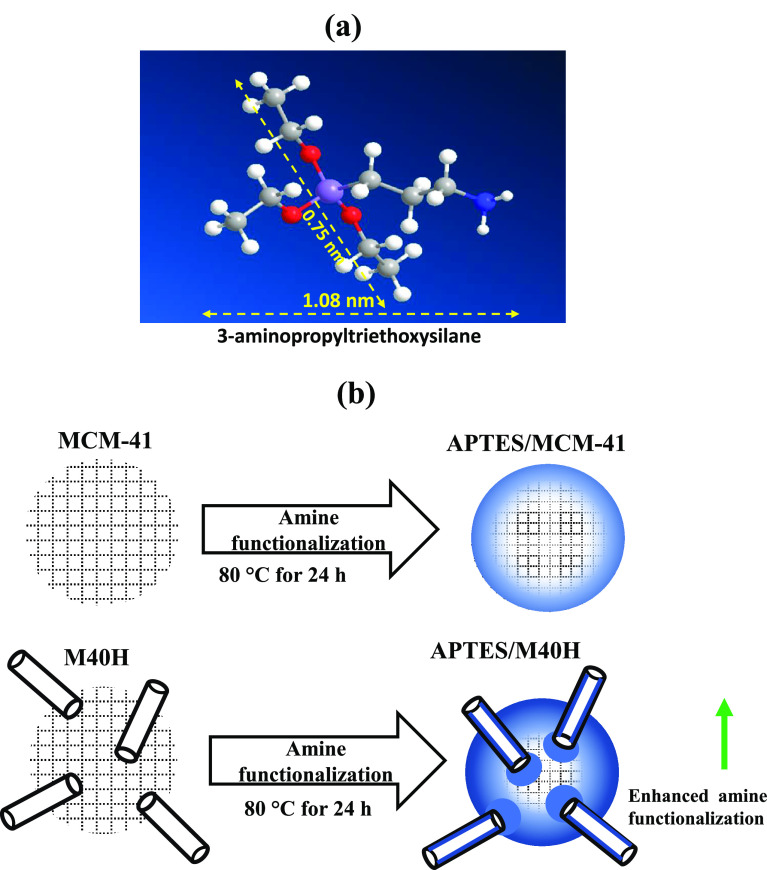
(a) Molecular structure and dimensions of the 3-aminopropyltriethoxysilane (APTES) molecule. (b) Schematic illustration of the concept of enhanced amine functionalization of MCM-41 using halloysite nanotubes as straws.

The focus of this work is to develop an improved MCM-41 type class II adsorbents by inserting nanostraws into the silica matrix to enhance the amine functionalization of the interior silica walls for improved CO_2_ capture.^9,10^ In MCM-41 the pores are 2–4 nm, while the amine moieties could occupy up to 2 nm of the pore dimension.^11^ For example, 3-aminopropyltriethoxysilane (APTES, Figure 1a) has molecular dimensions of a length of 1.08 nm and a transverse dimension of 0.75 nm. Functionalizing MCM-41 with APTES could lead to difficulties in complete loading, as the interior region near the external surface becomes saturated with the amine. Earlier work by Rao and co-workers^12^ found a saturation in CO_2_ adsorption capacity with increased functionalization of APTES, with thermogravimetric analysis showing about a 12 wt % loading of APTES. Similar results indicating pore blockage to full entry of APTES have been indicated in the literature.^13,14^ The tight fitting of amine-based polymers in the pores of MCM-41 therefore could create difficulties in fully utilizing MCM-41 particles for carbon capture, and it is possible that the interior of the particle is underutilized.

Our concept involves the integration of tubular nanostraws within the matrix of MCM-41 as an attempt to enhance access to the particle interior. These nanostraws are made with halloysite nanotubes. Halloysite nanotubes (HNTs) are naturally occurring aluminosilicate (Al_2_(OH)_4_Si_2_O_5_·*n*H_2_O) tubular materials with an anionic Si–O–Si surface and a cationic internal Al–OH surface. Depending on the source, halloysites have dimensions of 0.5–3 μm length, internal (lumen) diameter of 15–30 nm, and typically low specific surface areas <80 m^2^/g.^15^ Halloysite nanotubes have been explored as supports for class I and class II adsorbents^16−18^ due to the available lumen for amine encapsulation (class I) and ease of modification of its external surface for amine grafting (class II).

Figure 1b illustrates our concepts. We introduce HNT into MCM-41 as “nanostraws” with the hypothesis that the large pores of HNT will facilitate the entry of amines to the interior and thus improve amine loading. In our earlier work, we studied class I type systems of encapsulating polyethylenimine (PEI) and showed that the introduction of HNT into MCM-41 improved PEI loading by 40% and doubled CO_2_ capture efficiencies.^19^ In this study, we extend this concept of improved access to the particle interior through the nanostraws to enhance the covalent grafting of APTES into the MCM-41 matrix and develop an improved class II adsorbent with higher CO_2_ capture.

**Figure 2:**
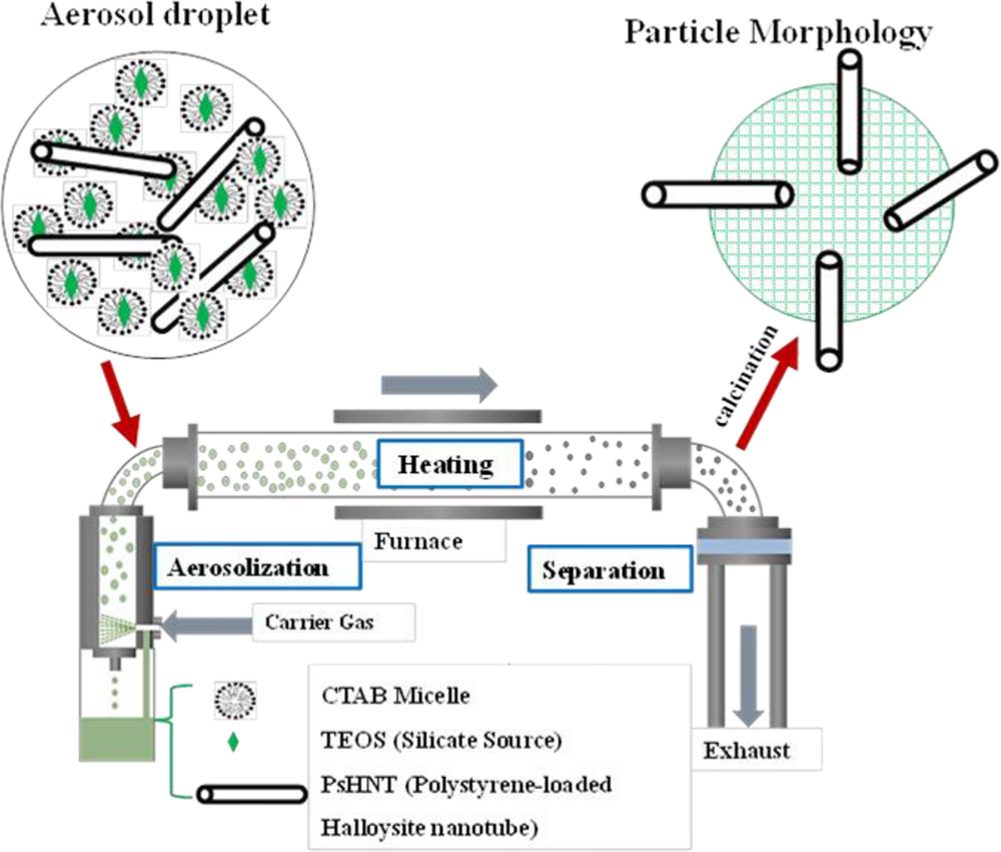
Illustration of the aerosol-assisted synthesis technique.

The method used to integrate HNT into MCM-41 is essentially based on a one-step aerosol-assisted synthesis where MCM-41 is synthesized in aerosol droplets^10,20,21^ that contain HNT (Figure 2). This approach is therefore based on chemistry within a droplet where MCM-41 is synthesized around HNT nanotubes, resulting in the morphology shown schematically in Figure 2. This paper describes our method to introduce HNT nanotubes into MCM-41 and the use of such composite particles in the capture of CO_2_. This procedure leads to particles with a hierarchical porosity which we evaluate in carbon capture and catalysis.^22^

## 2 Experimental Section

### 2.1 Materials

Hexadecyltrimethylammonium bromide (CTAB, 95%), tetraethyl orthosilicate (TEOS, 98%), hydrochloric acid (HCl, 37%), 3-aminopropyltriethoxysilane (APTES, 99%), toluene (99.5%, A.C.S. reagent grade), and polystyrene (PS, *M*_w_ 35000) were purchased from Sigma-Aldrich and used without any modifications. Halloysite nanotubes (HNT, average length of 1 μm and inner diameter of 18–25 nm) from Camel Lake, Australia, was received as a gift from John Keeling^23^ (Department of State Development–Geological Survey, South Australia). Deionized (DI) water with a resistance of 18.2 MΩ was procured from an Elga water purification system (Medica DV25).

### 2.2 Preparation of Polystyrene-Loaded Halloysite Nanotubes

The loading of polystyrene (PS) as a sacrificial hydrophobic polymer into the lumen of the HNT was achieved using a vacuum suction technique. First, 0.2 g of PS was dissolved in 10 mL of acetone and vigorously stirred for 15 min at room temperature, followed by the addition of 0.8 g of HNT. Magnetic stirring was continued for 3 h, and the resulting solution was transferred into a 25 mL round-bottom flask which was then attached to a rotavap. While rotating the round-bottom flask for continuous suspension of the PS-HNT solution, vacuum suction was applied at 150 mbar to facilitate the entry of PS into the HNT lumen and simultaneously evaporate the acetone solvent. Afterward, the sample was allowed to dry for 1 h at 10 mbar. The dried particles containing 20 wt % PS in HNT (PsHNT) were collected and stored.

### 2.3 Synthesis of MCM-41 and Composite Particle of Halloysite Nanotubes in MCM-41

The synthesis of MCM-41 was achieved using a one-step aerosol-assisted synthesis technique following a previously reported procedure with slight modifications.^20,24^ To this end, we prepared a single precursor solution comprising a solvent, a cationic surfactant template (CTAB), HNTs, and an organosilicate source. Briefly, 0.6 g of CTAB was added to 7.5 mL of 200 proof ethanol and sonicated for 5 min in a bath sonicator (Cole-Parmer 8890) for complete dissolution. While stirring the CTAB–ethanol solution, 1.5 mL of 0.1 M HCl was added followed by the dropwise addition of 2.25 mL of TEOS after 3 min. The addition of HCl to the MCM-41 precursor solution is to reduce the rate of siloxane condensation to enhance silica–CTAB self-assembly during the rapid aerosol-assisted synthesis process.^20^ The final precursor solution was rapidly transferred into a low-cost nebulizer (Micro Mist, Teleflex Inc., 1 mm orifice diameter). Atomization into aerosol droplets was achieved by bubbling N_2_ as a carrier gas through the precursor solution. As schematically illustrated in Figure 2, the aerosol droplets were conveyed through the orifice of the nebulizer into the heating zone at a carrier gas flow rate of 2.5 L/min. The heating zone comprises a 120 cm long quartz tube with an inner diameter of 5 cm tucked into a furnace of 76 cm length operating at 400 °C. A brief residence time of approximately 36 s was obtained based on the flow rate of the carrier gas and the dimensions of the furnace. At the other end of the furnace, a cellulose filter paper (Merck Millipore Ltd., pore size = 0.22 μm) was utilized to collect the dried and powdered particles from the heating zone. To prevent moisture condensation during collection, the filter paper was maintained at 80 °C by using heating tape. The accumulated particles were calcined at 550 °C at a heating rate of 5 °C/min in air to completely burn off the surfactant template.

The integration of HNT into MCM-41 was achieved by adding polystyrene-loaded HNT (PsHNT) into the precursor solution of CTAB and TEOS to achieve 40 wt % of HNT (M40H, based on silicon composition) in the composite. Briefly, 0.24 g of PsHNT was added to the CTAB solution (0.6 g of CTAB in 7.5 mL of ethanol) and bath sonicated for 30 s followed by stirring for 3 min to achieve homogeneous dispersion of the PsHNT in the solution. Afterward, 1.5 mL of 0.1 M HCl was added, followed by the dropwise addition of 2.25 mL of TEOS after 1 min. The final mixture was transferred into the nebulizer for aerosolization using the previously described procedure for MCM-41, with an increased flow rate of 4 L/min (22 s of residence time) to constantly suspend the PsHNT in the precursor solution. The obtained particles were calcined at 550 °C to remove the surfactant template and polystyrene to obtain 40 wt % of HNT in MCM-41 (M40H). It is worth noting that the use of polystyrene filled HNT was employed to ensure that the HNT lumen remained clear after the aerosol synthesis. In principle, CTAB adsorption to the HNT lumen should be minimal, as the surfactant and the lumen surface are cationic. Nevertheless, to prevent MCM-41 from forming within the lumen, we fill the lumen with the sacrificial polymer (polystyrene, PS) that is insoluble in the ethanol–water solvent used in the precursor solution. The morphological evidence of the PsHNT in MCM-41 is presented in the Supporting Information S-2. Upon calcination of the dried material, the polymer is removed together with surfactant in the pores of MCM-41, leaving empty HNT embedded in MCM-41.

### 2.4 Amine Functionalization of MCM-41 and M40H with APTES

The amine modification of MCM-41 and 40 wt % of HNT in MCM-41 (M40H) was achieved by covalent grafting of the amine functional group onto the mesoporous silica pore walls. This was achieved by adding 0.25 g of MCM-41 or M40H to 12.5 mL of toluene in a round-bottom flask under magnetic stirring for 20 min to achieve homogeneous dispersion. While stirring, 0.125–2.5 g of APTES was added in drops to the mixture and stirred for 20 min. The resulting mixture in the flask was transferred to an oil/water bath maintained at 80 °C and refluxed for 24 h. The mixture was separated by using a centrifuge and washed repeatedly with ethanol. The obtained sample was dried in air at 75 °C for 12 h. The sample nomenclature for APTES-functionalized MCM-41 is APTES/MCM-41_*X* and APTES-functionalized M40H is APTES/M40H_*X*, where
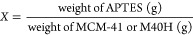
1

### 2.5 Materials Characterization

The structural analysis of the samples was performed using X-ray diffraction (XRD, Rigaku Miniflex II with a Cu Kα radiation at 1.54) at a small-angle scanning range of 2θ = 1.5°–7.0°. Using a nitrogen gas sorption technique (Micromeritics, ASAP 2010), textural analysis of porosity and surface area were estimated using the Brunauer–Emmett–Teller (BET) isotherm for evaluation of the surface area. The morphology of MCM-41 and M40H before and after amine functionalization was examined using scanning electron microscopy (Hitachi SEM-4800 field emission at 3 kV operating voltage) and transmission electron microscopy (TEM; FEI Tecnai G2 F30 twin transmission at 300 kV operating voltage). Bare and amine-modified samples were prepared for the cut section TEM by depositing each particle within epoxy resin followed by thin sectional cutting (100 nm thickness) using a diamond knife. The analysis of amine functional groups on the samples was done using Fourier transform infrared spectroscopy (FTIR; Thermo Scientific, NICOLET iS50R). Thermogravimetric analysis (TGA; TA Instruments Q500) was performed to quantify the composition of amine modification by APTES on each sample, over a temperature range of 100–710 °C.

### 2.6 CO_2_ Capture Studies

The thermogravimetric analysis (TGA) technique^25^ was used to study the CO_2_ adsorption capacity of amine-functionalized MCM-41 and M40H (40 wt % of HNT in MCM-41) at dry conditions. While APTES comprise only primary amines, the amino functional group covalently bonded to the silica walls of MCM-41 can capture CO_2_ molecules to form zwitterionic carbamates.^26,27^ For each study, approximately 10 mg of the amine-functionalized sample was loaded onto a dry platinum TGA pan and placed in the furnace. First, the sample was pretreated by heating to 105 °C at 5 °C/min for 1 h under N_2_ gas flow to completely remove any adsorbed gases or moisture from the surface of the particles. Next, the temperature was reduced to 35 °C followed by the introduction of pure dry CO_2_ gas at 90 mL/min for 2 h to adsorb CO_2_. The difference between the initial and final adsorbent weights after the introduction of CO_2_ gas was estimated as the weight of CO_2_ adsorbed by the sample.

## 3 Results and Discussion

### 3.1 Materials Characterization

**Figure 3:**
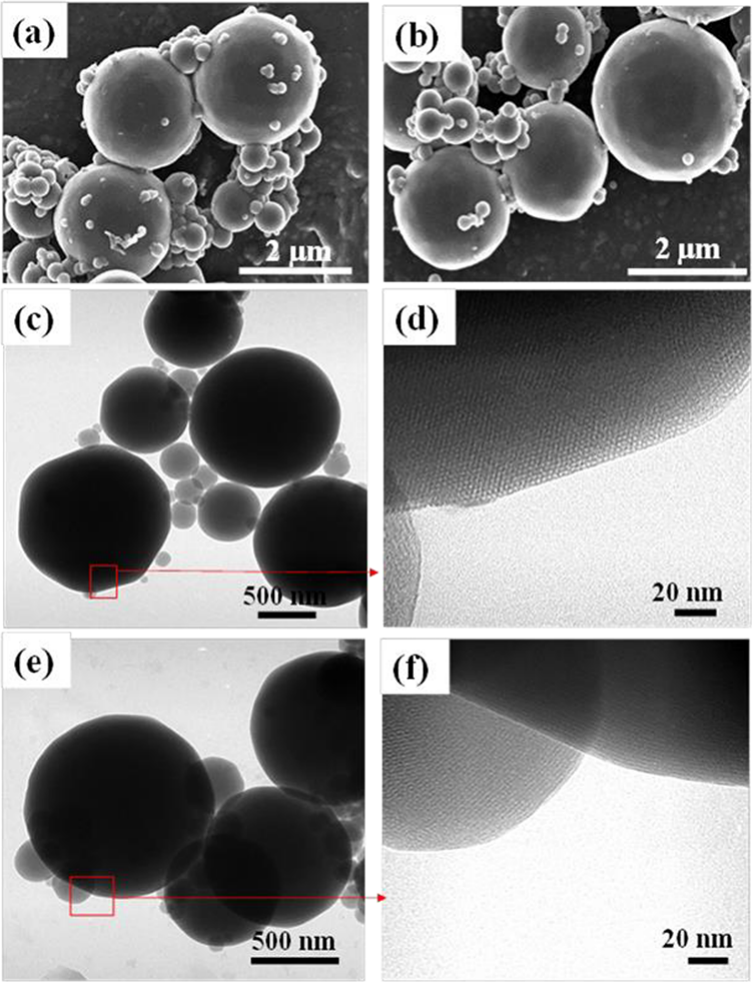
SEM image of (a) MCM-41 showing spherical polydisperse particles and (b) APTES/MCM-41_10 with similar MCM-41 morphology. TEM image of (c) MCM-41 and (d) high-resolution image of MCM-41 showing the presence of hexagonal arrangement of mesopores and (e) APTES/MCM-41_10 which shows no change in morphology relative to MCM-41. (f) High-resolution image of APTES/MCM-41_10.

Figure 3a shows SEM images of the control sample of MCM-41 as obtained through the aerosol-based process indicating spherical particles with polydisperse particle sizes ranging from 50 nm to ∼2 μm^21,28^ due to the large 1 mm orifice opening of the nebulizer.

**Figure 4:**
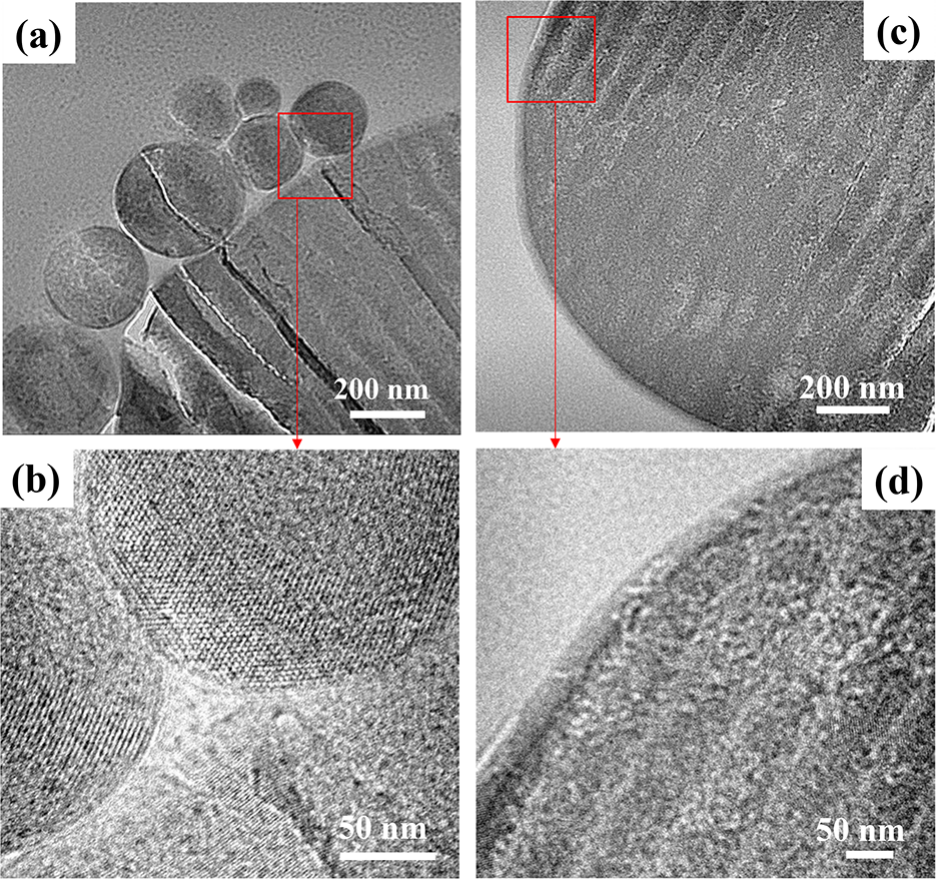
Cut-section TEM images of (a) MCM-41. (b) High-resolution image of MCM-41 showing the presence of ordered hexagonal array of pores and (c) APTES/MCM-41_10. (d) High-resolution image of APTES/MCM-41_10 showing presence of less visible MCM-41 pores.

Figure 3b shows essentially the same morphology upon amine modification. The corresponding TEM images at low and high resolutions are shown for the control (Figures 3c and 3d) and for the amine-modified sample (Figures 3e and 3f). It is very difficult to see clear distinctions between the control and the amine-modified MCM-41 (APTES/MCM-41_10) with direct TEM so we have attempted to image these using cut-section methods. Figure 4 illustrates clear differences between the control and the amine-functionalized sample. The ordered array of pores appears clearly visible in the high-resolution cut-section image of MCM-41 (Figure 4b), and on the contrary, the pores of APTES/MCM-41_10 (Figure 4d) are not visible. This observation supports pore occupancy by the grafted aminopropyl group (C_3_H_8_N) of APTES along the silica pore walls reducing the available pore space, in accordance with the results of Talavera-Pech and co-workers.^29^

**Figure 5:**
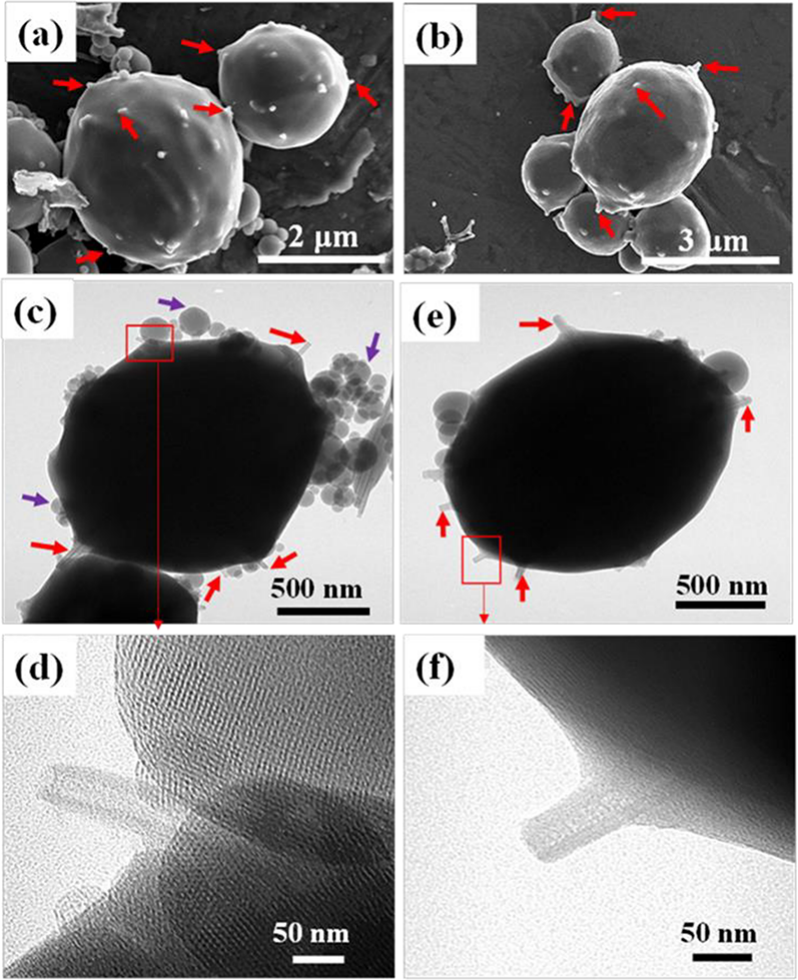
SEM image of (a) M40H which is obtained after the calcination of M40PsH and (b) amine-functionalized M40H (APTES/M40H_10). TEM image of (c) M40H. (d) High-resolution image of M40H showing the clear lumen of protruding HNT and hexagonal arrangement of MCM-41 pores after calcination and (e) APTES/M40H_10. (f) High-resolution image of APTES/M40H_10 showing the nanotube protrusions.

Figure 5 shows the morphology of MCM-41 particles containing halloysite nanotubes after complete calcination to remove organics, including CTAB from the MCM-41 and polystyrene from the HNT. TEM images of the precalcined materials are shown in the Supporting Information S-2 where we have preloaded the HNT with PS prior to the aerosol process to minimize the potential formation of MCM-41. The SEM images in Figure S-2a,b show the presence of HNT protrusions from the MCM-41 surface. As shown in the high-resolution TEM image in Figure S2d, the lumen of the protruded HNT appears to be blocked due to the presence of the loaded sacrificial polymer (PS) before calcination. As shown in the TEM images of the calcined samples in Figures 5c and 5e, the large composite particles (1.5–3.0 μm) have HNT protrusions as nanostraws (red arrows) with the presence of small satellite particles (purple arrows) which are entirely made of MCM-41. The high-resolution TEM image in Figure 5d shows the presence of an ordered mesoporous MCM-41 framework and possible evidence of a clear HNT lumen, signifying the removal of the sacrificial polymer from the HNT lumen after calcination. While the pores of the amine modified sample (APTES/M40H_10) are less visible, as shown in Figure 5f, the HNT lumen is still visible in the direct TEM image.

**Figure 6:**
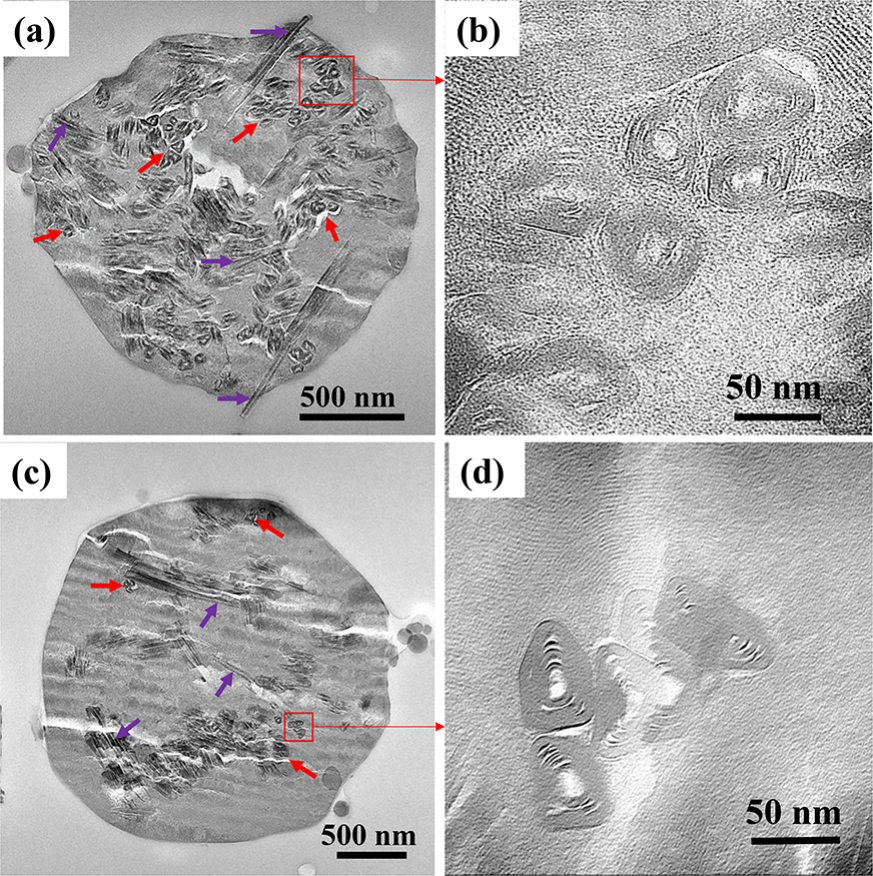
Cut-section TEM images of (a) M40H. (b) High-resolution image of M40H showing the cross section of HNT straws with clear lumen and (c) APTES/M40H_10. (d) High-resolution image of APTES/M40H_10 showing the cross section of the HNT lumen.

The cut-section TEM images of M40H (MCM-41 with a 40 wt % loading of HNT) and APTES/M40H_10 (amine-functionalized MCM-41 with a 40 wt % loading of HNT) are shown in Figures 6a and 6c. We note that the cut section leads to images of the HNT at multiple orientations, including those orthogonal to the section (red arrows of Figures 6a and 6c) and those partially longitudinally aligned to the section (purple arrows). The high-resolution TEM images in Figures 6b and 6d show the cross-sectional cuts of the nanostraws, and the significant decrease in contrast intensity indicates that the HNT lumen may be clear. The lumen is also visible in HNT that is oriented parallel or partially transverse. It is difficult to distinguish between the control and amine modified sample by visual examination of the cut-section TEMs because of the low electron density of the amines that may be present in the lumens of the amine-modified sample. Nevertheless, functionalization of the 20 nm of the lumen with APTES (∼1 nm) should leave the internal diameter significantly available for molecular transport due to its relatively large size compared to the size of the attached aminopropyl group from APTES.

**Figure 7:**
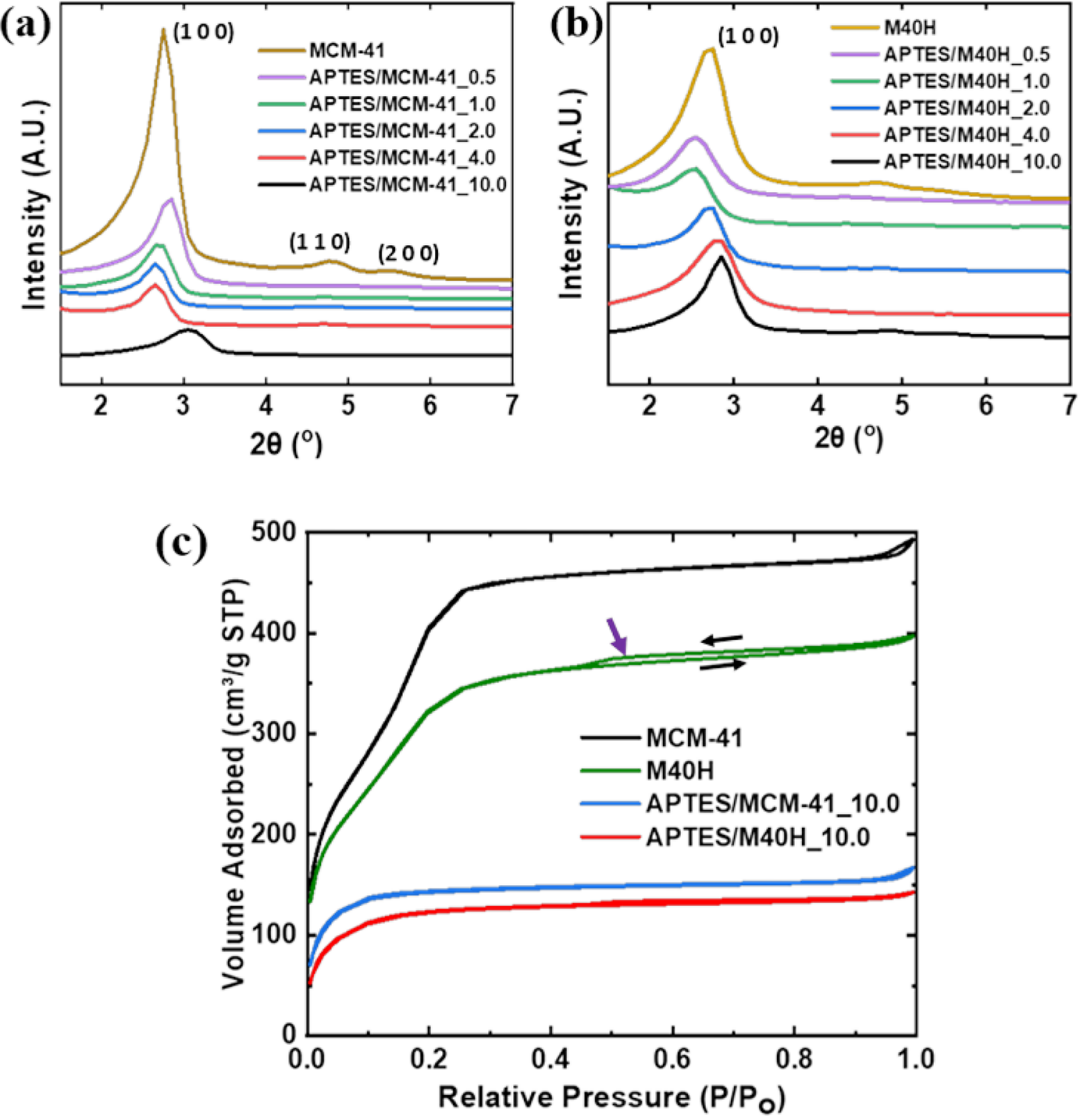
Powder X-ray diffraction (XRD) analysis of (a) MCM-41 and APTES-modified MCM-41 showing the presence of an intrinsic (100) MCM-41 peak. The intensity of the (100) peak reduces as the weight of APTES increases. (b) XRD analysis of M40H and APTES-modified M40H. (c) BET isotherm plot for MCM-41, M40H, APTES/MCM-41_10, and APTES/M40H_10.

The structural analysis of the control and modified samples is presented in Figure 7. As shown in Figures 7a and 7b, the XRD patterns for MCM-41 and M40H exhibit the characteristic (100) MCM-41 diffraction peak at 2θ = 2.75°, which corresponds to a *d*-spacing of 3.2 nm, and secondary (110) and (200) peaks at 2θ = 4.85° and 5.55°. The M40H (100) peak is broadened in comparison to the (100) peak for MCM-41 and is less intense due to the incorporation of 40 wt % HNT. We note that in the M40H system there are two populations of MCM-41: one being nucleated at the HNT interface due to CTAB binding to the external surface and the other that continues to grow out of this initial nucleation and is more symptomatic of MCM-41 in the bulk. These different populations may be responsible for the small differences observed for the MCM-41 XRD peaks for pristine MCM-14 and M40H. We also note that the introduction of APTES leads to small changes in peak position, which we attribute to the inclusion of Si atoms from APTES that results in a small but perhaps not negligible contribution to the X-ray scattering. This shows that the incorporation of HNT as straws into MCM-41 did not alter the mesoporous MCM-41 framework.^19,22^ Upon amine functionalization, the intensity of the (100) peak decreases as the weight of APTES increases and the secondary peaks lose resolution. This can be attributed to the attached APTES chain within the silica framework.^30−32^ The presence of silicon and oxygen in the organic APTES molecules increases the electron density of MCM-41 and M40H after amine functionalization. This leads to increased X-ray scattering within the amine-modified silica matrix^33^ which consequently reduces the XRD peak intensities of the amine modified samples compared to the bare MCM-41 and M40H. However, we note that further study (perhaps using a high-flux SAXS instrument) is needed. The presence of the intrinsic (100) MCM-41 peak shows that the meso-structure of the MCM-41 is maintained after amine functionalization.^29,34,35^

As summarized in Table 1, MCM-41 exhibits a high surface area of 1418 m^2^/g. The integration of HNT nanostraws with a significantly low specific surface area (50 m^2^/g)^36,37^ leads to a reduced but still relatively high surface area of 1097 m^2^/g for M40H, which roughly corresponds to the weighted surface areas of MCM-41 and HNT in the composite. The high surface area of M40H supports the evidence from XRD that the integration of HNT with tubular morphology into spherical MCM-41 particles has no effect on the mesoporous framework and structure of MCM-41. As shown in Figure 7c, the BET isotherm of M40H shows a small hysteresis. This can be attributed to the incorporation of HNT acting as a large mesopore within the MCM-41 framework containing narrow pores (2–4 nm). This leads to the generation of hierarchical mesopores which consequently leads to the formation of a type IV isotherm and hysteresis loop which generates multilayer adsorption of probe N_2_ molecules and capillary condensation.^18,22,38,39^ Amine functionalization of MCM-41 and M40H leads to a significant reduction in BET surface areas due to amine grafting.^30,32,34^

**Table 1:** BET Surface Area Analysis of Bare and Amine-Modified Samples

sample	BET surface area (m^2^/g)
MCM-41	1418
HNT	49
M40H	1097
APTES/MCM-41_10	432
APTES/M40H_10	382

**Figure 8:**
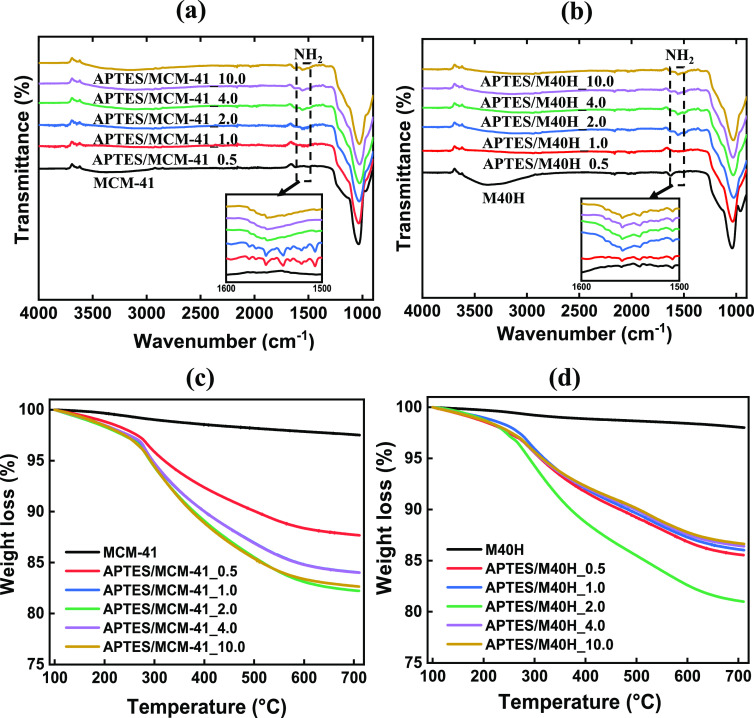
FT-IR analysis of (a) MCM-41 and amine-functionalized MCM-41 samples and (b) M40H and APTES-functionalized M40H samples. The presence of the NH_2_ stretching band at ∼1560 cm^–1^ (insets showing expanded wavenumber 1500–1600 cm^–1^) for the amine-modified samples confirms the presence of an amine functional group. TGA weight loss analysis showing the quantification of amines on (c) MCM-41 and (d) M40H.

The presence of the amine functional group on all modified samples was also confirmed by Fourier transform infrared (FT-IR) spectroscopy analysis. As shown in Figures 8a and 8b, both MCM-41 and M40H exhibit absorption bands characteristic of silica materials.^40,41^ Relative to the bare MCM-41 and M40H samples, all amine-functionalized samples of MCM-41 and M40H exhibit the distinct appearance of the NH_2_ bending vibration at 1560 cm^–1^, which confirms the presence of the amine functional group. It is worth noting that the low intensity of the NH_2_ absorption band is due to the low amine density of alkoxysilanes such as APTES compared to polymeric amines like polyethylenimine (PEI).^42^ Additionally noted is the disappearance of the O–H stretching at adsorption band ∼3300 cm^–1^ after functionalization with APTES due to the reaction of silanol groups with the ethoxy group of APTES.^32,34,43^

The amount of covalently attached aminopropyl groups was quantified using weight loss estimation from thermal degradation of the pristine and amine-modified MCM-41 and M40H samples. As shown in Figure 8c, MCM-41 exhibits a good stability with a gradual weight loss due to the loss of surface silanol groups.^44^ For the amine-functionalized samples, the weight loss at a temperature range of 100–275 °C is due to the loss of adsorbed water and degradation of functionalized APTES. Decomposition of the grafted organic aminopropyl group of APTES occurs primarily above 275 °C leading to a rapid loss of weight.^29,30,44^ Quantitatively, the modified samples exhibit weight loss from 12.31–17.77%, with APTES/MCM-41_2.0 exhibiting the highest weight loss. This shows that beyond an APTES loading of *X* = 2.0, there is no further uptake of APTES, and excess APTES is removed through washing. In Figure 8d, the addition of 40 wt % HNT (M40H) with amine modification shows a weight loss of 13.36–19.0% as *X* increases from 0.5–10.0. Of these samples, APTES/M40H_2.0 exhibited the highest weight loss. Increasing the APTES initial loading beyond *X* = 2.0 appears to decrease grafting levels, perhaps due to the fact that APTES saturation at the HNT tips prevents further entry of APTES into the particle. Our hypothesis is that at high loading the bolus of APTES causes accumulation both on the MCM-41 surfaces and at the tips of the HNT. The accumulation reduces the APTES that can enter into the particle and be functionalized, leading to an observed maximum in functionalization at *X* = 2. Our results on CO_2_ capture presented next appear to verify this possibility.

### 3.2 CO_2_ Capture Studies and Kinetics

Our concept is based on the incorporation of HNT nanostraws into mesoporous MCM-41 particles to promote enhanced diffusion of molecular species. To prove that this concept works, we evaluated the capture of CO_2_ via adsorption into the APTES-modified MCM-41 or M40H (HNT containing MCM-41) particles. This was done at 35 °C and atmospheric pressure, and the CO_2_ molecules are captured by chemisorption to form carbamates in dry conditions,^26^ according to eqs 2 and 3.
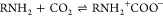
2
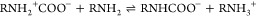
3

**Figure 9:**
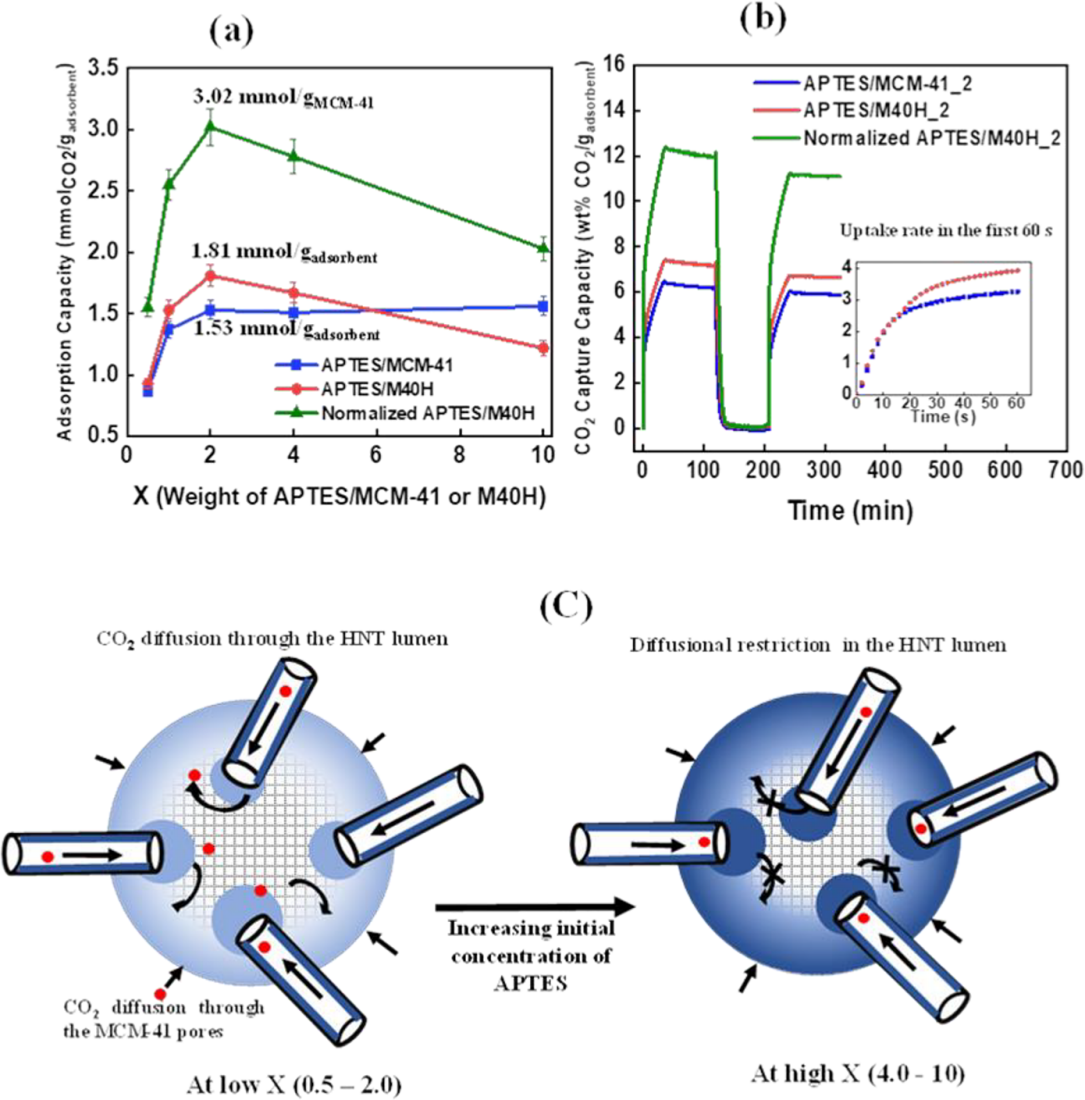
(a) CO_2_ adsorption capacity plot showing the change in adsorption capacity of APTES/MCM-41, APTES/M40H, and normalized APTES/M40H as *X* increases. (b) CO_2_ adsorption–desorption kinetics for APTES/MCM-41, APTES/M40H, and normalized APTES/M40H at *X* = 2. The inset shows the initial uptake rate for APTES/MCM-41 and APTES/M40H within the first 60 s of introducing CO_2_ gas. (c) Proposed illustration of diffusional restriction in the amine-functionalized M40H as the concentration of APTES increases. The black arrows show the diffusion of the CO_2_ molecules.

Figure 9a shows the adsorption capacity for APTES/MCM-41 and APTES/M40H as a function of the loading of APTES. The APTES/MCM-41 saturates out in an adsorption capacity (1.53 mmol_CO_2__/g_adsorbent_) after a loading of 0.5 g (*X* = 2). Further increment in the weight of APTES shows no effect on adsorption capacity of APTES/MCM-41. Thus, we can posit the possible saturation of MCM-41 with amine functional groups at APTES weight of 0.5 g, consequently leading to pore blockage. Remarkably, APTES/M40H exhibits a 20% higher CO_2_ capture performance than APTES/MCM-41 at an APTES loading of 0.125–1.0 g (*X* = 0.5–4.0) with an optimal adsorption capacity of 1.81 mmol_CO_2__/g_adsorbent_ at *X* = 2.0 (0.5 g of APTES). This observation supports the hypothesis of enhanced amine functionalization of silanol sites. Furthermore, the HNT lumen could serve as a larger channel for diffusion of CO_2_ molecules into the interior amine sites of MCM-41 as shown by the curved arrows of Figure 9c (left). At APTES loading >0.5 g, the adsorption capacity of APTES/M40H starts to drop. This may be explained through the illustrative schematics in Figure 9c which indicate amine saturation and pore blockage at the tips of the HNT and difficulty of CO_2_ accessing the interior amine layer (Figure 9c, right). We note that the CO_2_ adsorption capacity when normalized on the basis of MCM-41 which has a surface area of 1418 m^2^/g far in excess of HNT (49 m^2^/g) shows that the composite system has an adsorption capacity significantly greater than that of MCM-41 alone (green curve of Figure 9a). This may clearly indicate improved functionalization of MCM-41 and improved access of CO_2_ to the particle interior.

The CO_2_ adsorption–desorption isotherms at *X* = 2.0 (0.5 g of APTES), the optimal uptake level, are shown in Figure 9b. A close observation of the uptake slopes at the initial stage (inset, Figure 9b) shows that both APTES/MCM-41 and APTES/M40H adsorbents have an essentially similar initial rate of adsorption with a continuation in adsorption to a higher saturation level in the M40H system, as CO_2_ can diffuse through the tubes and access the interior of the particle. It is evident that the incorporation of HNT increases the CO_2_ adsorption capacity of amine-functionalized MCM-41, and the retention of capture performance over 5 multiple cycles shows the potential stability of the adsorbent (Figure S3). The increase in the level of CO_2_ capture shows that the introduction of HNT does not damage the properties of the class II adsorbent to adsorb CO_2_ molecules. Table 2 shows the adsorption capacity of the adsorbents normalized based on the surface area of the pristine samples, and this follows the same trend shown in Figure 9a.

**Table 2:** Normalized CO_2_ Adsorption Capacity Based on the Surface Area of the Bare Supports^a^

*X*	normalized APTES/MCM-41 (mmol_CO_2__/m^2^) × 10^–3^	normalized APTES/M40H (mmol_CO_2__/m^2^) × 10^–3^
0.5	0.61	0.66
1.0	0.97	1.08
2.0	1.08	1.28
4.0	1.07	1.18
10.0	1.10	0.86

a The MCM-41 and M40H support surface areas are 1418 and 1097 m^2^/g, respectively. *X* = weight of APTES (g)/weight of MCM-41 or M40H (g).

## 4 Conclusions

This work elucidates an approach to improve the CO_2_ adsorption capacity of amine-functionalized MCM-41 particles by inserting halloysite nanotubes to facilitate improved molecular transport. The integration of the nanotubes into MCM-41 is achieved using a one-step aerosol-assisted synthesis method to obtain a composite morphology of nanostraw protrusions from the surface of MCM-41. The obtained MCM-41 and composite M40H particles are subjected to amine functionalization with APTES to create a solid class II adsorbent that captures CO_2_ through chemisorption. Structural and textural analysis results show that the MCM-41 framework is maintained on all particles after amine modifications. While the covalent grafting of the organic amine group from APTES leads to pore blockage of the 2–4 nm MCM-41 pores, the tubular straws in the composite M40H particle improve the accessibility of the particle interior by transport through the lumen, potentially allowing a greater grafting density of APTES and leading to a 20% increase in CO_2_ adsorption based on the composite and a doubling of CO_2_ adsorption when normalized based on the surface area of MCM-41. However, the adsorption tends to drop at high APTES loadings, indicating possible pore blockage at the tips of the HNT nanotubes limiting further functionalization and the accessibility of interior amine sites to CO_2_.

The concept of halloysite-based nanostraws in mesoporous particles can therefore be effectively utilized to improve the diffusion of amine moieties into high surface area materials for enhanced amine functionalization and carbon capture. This method of the creation of hierarchical porosities is facile and does not reduce the structural characteristics of the porous matrix that are inherent in alternative etching methodologies. The concept is general and, in principle, can be adapted to a wide range of catalytic and adsorption technologies where access to the particle interior is hindered through diffusional restrictions.

## Supporting Information Available

Section S-1: SEM and TEM images for bare and polystyrene-loaded halloysite nanotube (PsHNT); Section S-2: the SEM and TEM of MCM-41 containing 40 wt % of PsHNT (M40PsHNT) before calcination; Section S-3: the adsorption–desorption plot of CO_2_ capture over 5 cycles and amine efficiencies (PDF)
