# Supplementary material for: Design of Nanostraws in Amine-Functionalized MCM-41 for Improved Adsorption Capacity in Carbon Capture
Source: Energy Fuels. 2023 Jul 26;37(16):12079–88. doi: 10.1021/acs.energyfuels.3c01318 (PMC10441579; doi:10.1021/acs.energyfuels.3c01318)
Supplement: Supplementary file 1 — ef3c01318_si_001.pdf [file ef3c01318_si_001.pdf]

SUPPORTING INFORMATION

Design of Nano-straws in Amine Functionalized MCM-41 for Improved Adsorption Capacity in Carbon Capture

Oluwole Ajumobi[1], Borui Wang[1], Azeem Farinmade[1], Jibao He[2], Julia A. Valla[3], Vijay T. John[1*]*

1. Department of Chemical & Biomolecular Engineering, Tulane University, 6823 St. Charles Avenue, New Orleans, Louisiana 70118, United States.
2. Coordinated Instrumentation Facility, Tulane University, 6823 St. Charles Avenue, New Orleans, Louisiana 70118, United States.
3. Department of Chemical & Biomolecular Engineering, University of Connecticut, Storrs, Connecticut 06269, United States.

Submitted to Energy & Fuels

*Corresponding authors

Vijay T. John – vj@tulane.edu

Julia A. Valla – Ioulia.valla@uconn.edu

S-1: Morphological Characterization of Pristine and Polystyrene-loaded Halloysite Nanotubes

The morphology of pristine halloysite nanotubes (HNT) and polystyrene (Ps) loaded HNT (PsHNT) is visualized with electron microscopy imaging. The SEM image of bare halloysite nanotubes (HNT) in **Figure S-1a** shows the expected tubular orientation with no change in the external surface after preloading the lumen with polystyrene (**Figure S-1b**). The TEM image in **Figure S-1c** shows the tubular structure of the bare HNT with an average length of 0.5 – 1.5 μm and visible lumen diameter of 15 - 20 nm. Comparatively, the lumen of the PsHNT appears to be blocked due to the presence of physically immobilized polystyrene, as shown in **Figure S-1d**.

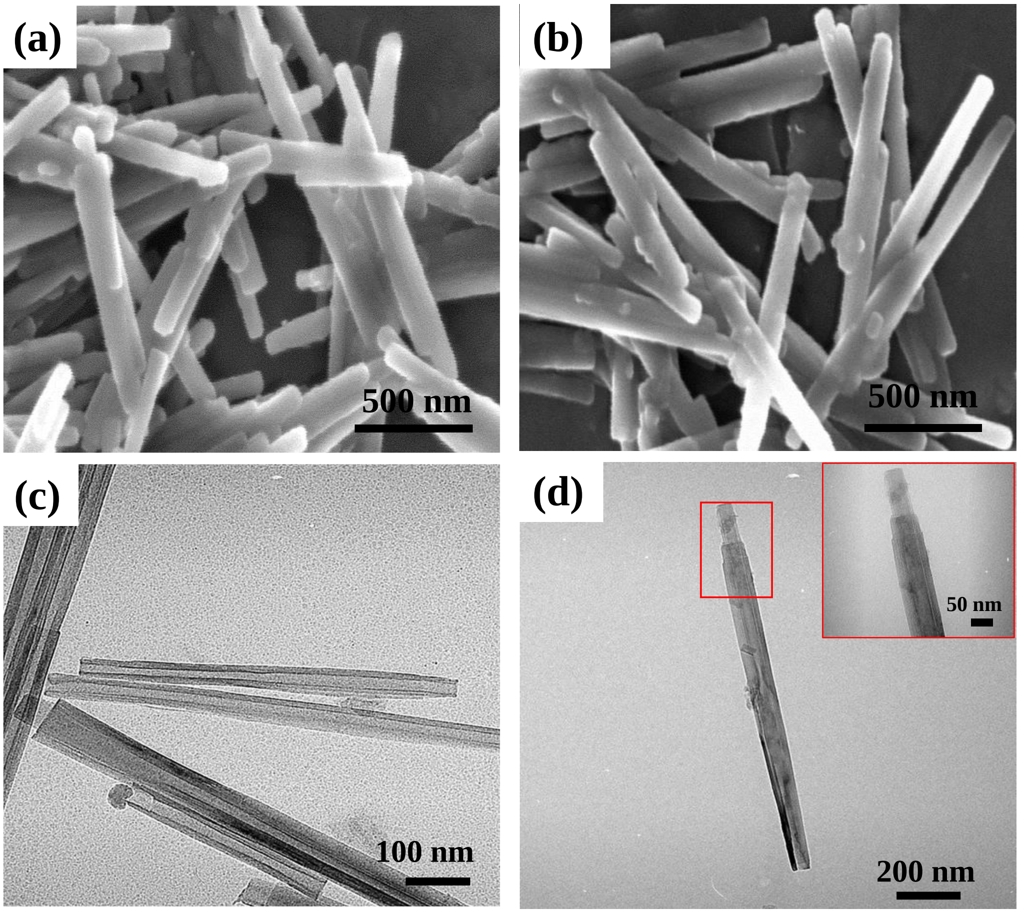

Figure S-1. SEM image of (a) bare HNT (b) PsHNT which shows minimal to no presence of polystyrene (PS) on the external surface of the HNT. TEM image of (c) pure HNT which shows the available lumen with both ends opened (d) Polystyrene loaded HNT showing the filled/blocked HNT lumen with PS.

S-2: MCM-41 containing of Polystyrene-loaded Halloysite Nanotubes

Figure S-2 shows the morphology of polystyrene-loaded HNT (PsHNT) in MCM-41 (M40PsHNT) before calcination. The SEM images in **Figure S-2a and b** show the presence of multiple protrusions of PsHNT from the surface of large MCM-41 particles. The PsHNT protrusion in the high-resolution TEM image shown in **Figure S-2d** appears to be blocked and this proves the presence of loaded PS within the lumen of the HNT. This shows that we have successfully infiltrated the HNT lumen with a hydrophobic polymer (polystyrene) and these polymer-filled HNT have been integrated into MCM-41 without losing the preloaded polymer during the preparation of the precursor solution and the aerosol synthesis.

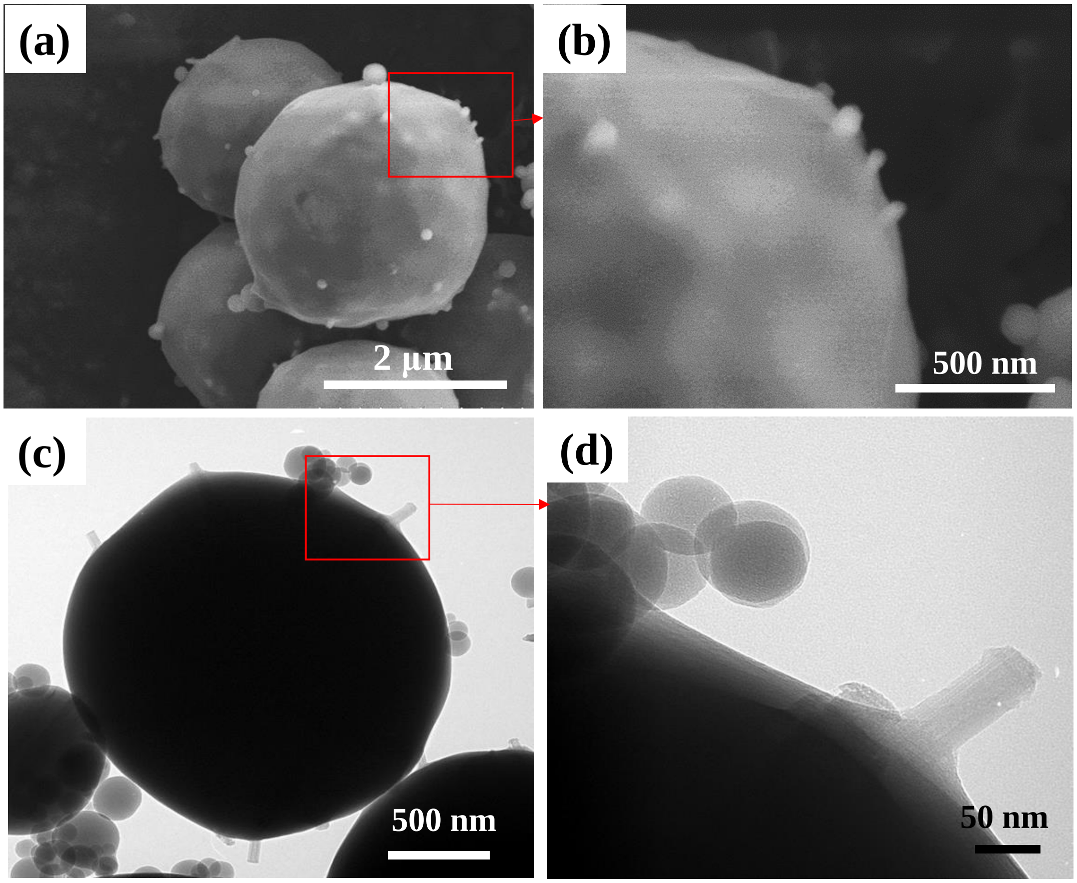

Figure S-2. SEM image of (a) M40PsHNT which shows protrusion of polystyrene-filled HNT (PsHNT) in MCM-41 (b) high resolution image showing the protrusion of PsHNT. TEM image of (c) M40PsHNT (d) high resolution image of M40PsH showing the lumen of HNT filled with polystyrene after aerosol synthesis.

S-3: CO_2 Adsorption-Desorption Cycles

Figure S-3 shows the adsorption-desorption plot of CO_2 capture over 5 cycles using APTES/MCM-41 and APTES/M40H adsorbents at APTES weight of 0.5 g (X = 2). The normalized APTES/M40H plot is generated based on 60 wt% of MCM-41 in the M40H composite (40 wt% of HNT in MCM-41) and has a capture capacity of 13.3 wt%CO_2/$g_{adsorbent}$ which doubles that of APTES/MCM-41 (6.72 wt%CO_2/$g_{adsorbent}$).

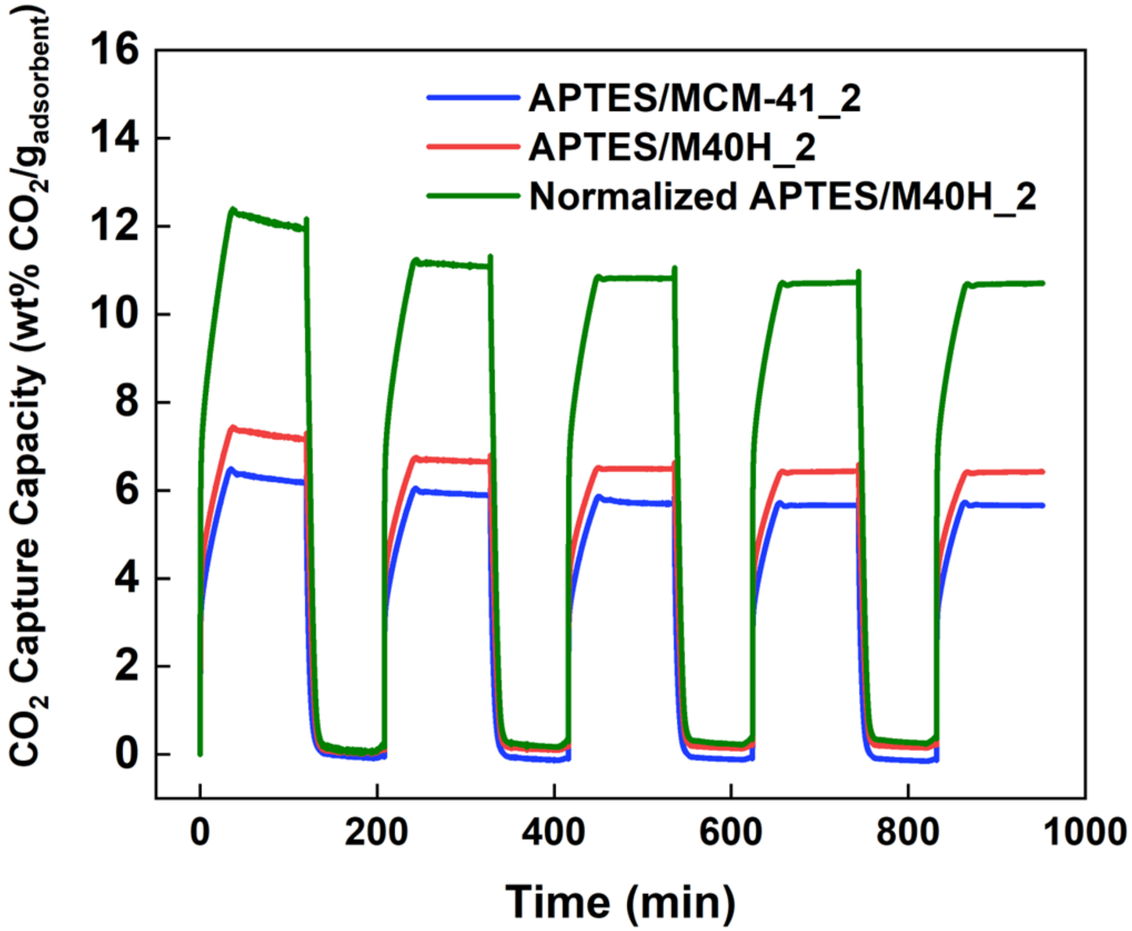

Figure S-3. CO_2 adsorption-desorption cycles over APTES/MCM-41, APTES/M40H, and normalized APTES/M40H.

The adsorption capacity and amine efficiencies of the amine functionalized MCM-41 and M40H adsorbents are summarized in **Table S1**. The amine content of each adsorbent is estimated using the TGA weight loss of each adsorbent as the amount of bonded aminopropyl group (C_3H_8N).

Table S1. Summarized adsorption capacity and amine efficiency of the amine adsorbents

**Samples**	**Adsorption Capacity ($mmol\ CO_2/g_{adsorbent}$)**	**Amine Content (mmol N/g)**	**Amine Efficiency ($mol\ CO_2/mol\ N$)**
**APS/MCM41_0.5**	0.87	1.69	0.51
**APS/MCM41_1**	1.37	2.32	0.59
**APS/MCM41_2**	1.53	2.63	0.58
**APS/MCM41_4**	1.51	2.32	0.65
**APS/MCM41_10**	1.55	2.56	0.61
**APS/M40H_0.5**	0.93	2.15	0.43
**APS/M40H_1**	1.53	2.06	0.74
**APS/M40H_2**	1.81	2.93	0.62
**APS/M40H_4**	1.67	2.00	0.83
**APS/M40H_10**	1.22	1.96	0.62
